# Amphibian population genetics in agricultural landscapes: does viniculture drive the population structuring of the European common frog (*Rana temporaria*)?

**DOI:** 10.7717/peerj.3520

**Published:** 2017-07-11

**Authors:** Patrick P. Lenhardt, Carsten A. Brühl, Christoph Leeb, Kathrin Theissinger

**Affiliations:** Institute for Environmental Science, Universität Koblenz-Landau, Germany

**Keywords:** Landscape genetics, Microsatellites, Amphibians, Common frog, Isolation by distance, Agriculture

## Abstract

Amphibian populations have been declining globally over the past decades. The intensification of agriculture, habitat loss, fragmentation of populations and toxic substances in the environment are considered as driving factors for this decline. Today, about 50% of the area of Germany is used for agriculture and is inhabited by a diverse variety of 20 amphibian species. Of these, 19 are exhibiting declining populations. Due to the protection status of native amphibian species, it is important to evaluate the effect of land use and associated stressors (such as road mortality and pesticide toxicity) on the genetic population structure of amphibians in agricultural landscapes. We investigated the effects of viniculture on the genetic differentiation of European common frog (*Rana temporaria*) populations in Southern Palatinate (Germany). We analyzed microsatellite data of ten loci from ten breeding pond populations located within viniculture landscape and in the adjacent forest block and compared these results with a previously developed landscape permeability model. We tested for significant correlation of genetic population differentiation and landscape elements, including land use as well as roads and their associated traffic intensity, to explain the genetic structure in the study area. Genetic differentiation among forest populations was significantly lower (median pairwise *F*_ST_ = 0.0041 at 5.39 km to 0.0159 at 9.40 km distance) than between viniculture populations (median pairwise *F*_ST_ = 0.0215 at 2.34 km to 0.0987 at 2.39 km distance). Our analyses rejected isolation by distance based on roads and associated traffic intensity as the sole explanation of the genetic differentiation and suggest that the viniculture landscape has to be considered as a limiting barrier for *R. temporaria* migration, partially confirming the isolation of breeding ponds predicted by the landscape permeability model. Therefore, arable land may act as a sink habitat, inhibiting genetic exchange and causing genetic differentiation of pond populations in agricultural areas. In viniculture, pesticides could be a driving factor for the observed genetic impoverishment, since pesticides are more frequently applied than any other management measure and can be highly toxic for terrestrial life stages of amphibians.

## Introduction

The survival of amphibian wildlife populations is threatened by habitat loss, fragmentation of populations, diseases, invasive species, climate change and toxic substances (Stuart et al., 2008). Underlying causes of habitat loss, fragmentation and habitat pollution with toxic substances are the expansion and intensification of agriculture (Gallant et al., 2007; Hartel et al., 2010) as well as built-up areas due to the development of traffic infrastructure, urbanization and industrialization (Löfvenhaft, Runborg & Sjögren-Gulve, 2004). While the hazard of built-up areas for amphibians is obvious (i.e., roads with car traffic as physical barriers), the threat of agriculture is more complex. Beside habitat loss and fragmentation of remaining suitable habitats or populations, agriculture often requires the development of irrigation, drainage and/or retention systems, which can impact the availability and quality of amphibian breeding sites. Yet despite their limited dispersal capacity compared with other vertebrates (Hillman et al., 2014), amphibians have been able to persist in agricultural landscapes by adapting to the altered availability of breeding sites (Mann et al., 2009). In agricultural landscapes, breeding habitats are often completely surrounded by arable land (Berger, Pfeffer & Kalettka, 2011). Thus, amphibians regularly have to cross agricultural land during dispersal and seasonal migration (i.e., spring migration for reproduction) or for foraging and are therefore likely exposed to field cultivation measures (Becker et al., 2007; Lenhardt, Brühl & Berger, 2014; Joseph, 2016).

The expansion and intensification of agriculture also involves input of a wide variety of agrochemicals into the environment. Pesticides play a crucial role in this context, since they can be highly toxic to terrestrial life stages of amphibians (Brühl et al., 2013; Cusaac et al., 2016). Additionally, a spatio-temporal overlap of pesticide applications with the terrestrial activity phase of amphibians was demonstrated for some crops (Lenhardt, Brühl & Berger, 2014). In a terrestrial exposure scenario, application-relevant rates of fungicides caused mortality rates of approximately 70% (Belden et al., 2010) and 100% (Brühl et al., 2013) of amphibian test organisms. Also, the use of two or more pesticides in a mixture application is very common and may cause higher toxicity compared to non-mixture applications (Kumar, 2014; Brodeur et al., 2014). Furthermore, pesticides from different applications may accumulate in surface waters (Ulrich et al., 2015), exposing adult amphibians and their larvae to a diverse pesticide mixture. The demonstrated sublethal and lethal toxicity of various pesticides on aquatic and terrestrial life stages of amphibians (Sparling & Fellers, 2009; Relyea, 2011; Denoël et al., 2013; Ghose et al., 2014; Lau, Karraker & Leung, 2015) suggests a potentially strong selection effect on meta-populations in agricultural landscapes. Furthermore, mortality or reduced locomotion capacity of amphibians due to pesticide exposure may promote the fragmentation of breeding pond populations (Lenhardt et al., 2013).

An indirect method to assess the effect of fragmentation on amphibian breeding pond populations is the use of neutral molecular markers, such as polymorphic microsatellites, i.e., non-coding DNA sequences consisting of tandem repeats and exhibiting high mutation rates (Jehle & Arntzen, 2002). By combining several microsatellite markers it is possible to estimate genetic differentiation among adjacent populations (Beebee & Rowe, 2008). Linear barriers, such as roads or major rivers, can cause a significant increase of genetic differentiation among amphibian breeding populations (Arens et al., 2007; Marsh et al., 2007). If agricultural fields function similarly as migration barriers or sink habitats, a population differentiation within a meta-population could be expected.

In the present study, we analyzed the genetic differentiation of six *Rana temporaria* LINNAEUS 1758 (European common frog) breeding pond populations from a viniculture landscape, using ten polymorphic microsatellite loci. Also, we analyzed four populations from the adjacent Palatinate Forest as a reference for widely unhindered gene flow. We tested for significant correlation of genetic population differentiation and landscape elements, including land use and linear barriers (roads and their associated traffic intensity), to explain the genetic structure in the study area. If viniculture acts as a migration limiting barrier for amphibians, we would reject the null hypothesis of a meta-population in the study area and rather expect a detectable genetic structuring among the analyzed *R. temporaria* breeding pond populations. Also, we compared the estimated genetic differentiation with the results of a landscape permeability model from the same study area (Lenhardt et al., 2013). In this model, pesticides were considered to decrease the permeability of agricultural land, causing a fragmentation or even isolation of amphibian breeding sites. The aim of the present study was to test the model predictions for the common frog by applying landscape genetic methods, i.e., whether the genetic differentiation of the examined breeding pond populations would reflect the predicted population fragmentation of common frogs in the vinicultural landscape.

## Material and Methods

**Figure 1:**
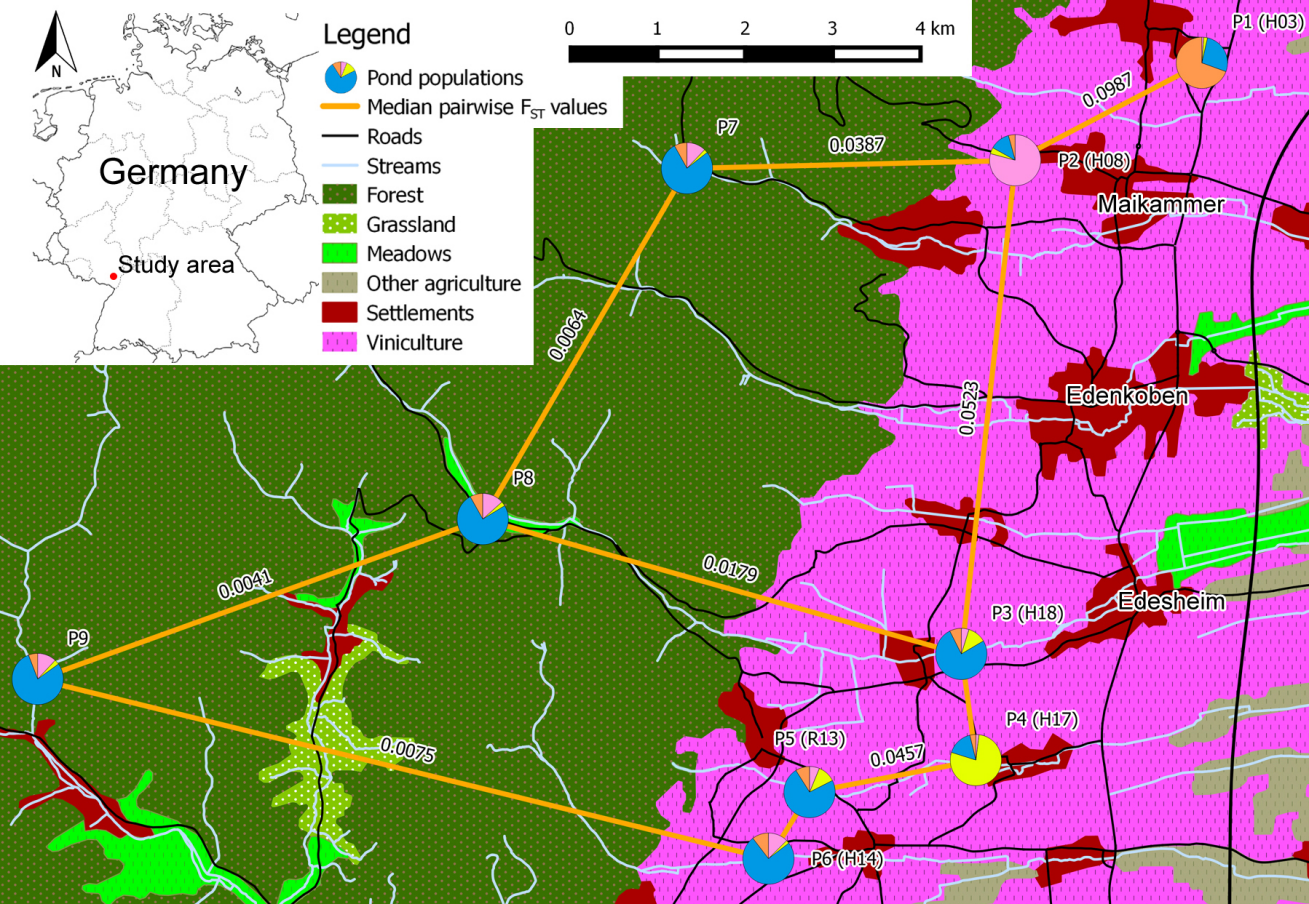
Schematic overview of the core study area in southern palatinate between “Neustadt an der Weinstraße” (north of P1) and “Landau in der Pfalz” (south of P6) with median pairwise *F*_ST_ values for selected pond population pairs. Pond labels of Lenhardt et al. (2013) in brackets. Pie charts of the pond populations show the overall share of each cluster on the population, based on the STRUCTURE analysis for clusters *K* = 4 (see Fig. 2 for cluster colors in pie charts).

The study was conducted in Rhineland-Palatinate, Germany, between Neustadt/Weinstrasse and Landau/Pfalz (Fig. 1; Figs. S1–S3). We sampled ten breeding pond populations of *R. temporaria* during the breeding seasons 2012–2014. Six of these ponds (P1–P6) were located in the vineyards of Southern Palatinate and four (P7–P10) were inside the adjacent Palatinate Forest. The distance between the sampled ponds P1–P9 varied between about 0.9 and 15 km, whereas P10 was located about 40 km northwest of the core study area near Kaiserslautern (Figs. S4). The waterbodies of breeding pond populations P3, P5 and P6 were directly connected to the Palatinate Forest by permanent or seasonal streams, whereas for P1, P2 and P4 this was not the case.

We collected eggs from all explicit distinguishable clutches (*N* = 7–10) of *R. temporaria* per breeding pond (P1–P9; in total 71 clutches) and hatched them in 300 ml glass bottles filled with tap water to Gossner stages 20–25. Sampling was approved by the Structure and Approval Directorate South of Rhineland-Palatinate, department 42, Upper nature conservation authority (approval number 42/553-254). Three tadpoles per clutch were randomly selected for genetic analysis. Since females of *R. temporaria* typically lay a single clutch per breeding season (Schlüpmann et al., 1996), we assumed only full-siblings existed within clutches. Furthermore, we included genetic data of 21 adult *R. temporaria* from P10 from a previous study (Müller, Lenhardt & Theissinger, 2013). We applied a high salt DNA extraction protocol to obtain DNA from tissue samples of the tadpoles (Aljanabi & Martinez, 1997).

We analyzed ten variable microsatellite loci (Table 1; Matsuba & Merilä, 2009) and amplified the fragments in two multiplex PCRs using the QIAGEN Multiplex PCR Kit (Hilden, Germany) following Müller, Lenhardt & Theissinger (2013). The selected loci were chosen from a number of tested loci due to their amplification success and polymorphism in an earlier study (Müller, Lenhardt & Theissinger, 2013). Also, six of the selected loci were located on different chromosomes (Table 1; see Cano et al., 2011). Amplification products were run on a CEQ 8000 Sequencer (Beckman Coulter, Krefeld, Germany). Fragments were analyzed with the software GeneMarker 1.95 (SoftGenetics, State College, Pennsylvania, USA) and verified with Micro-Checker 2.2.3 (Van Oosterhout et al., 2004).

**Table 1:** Basic information on used microsatellites: amplification success (AS) based on all data as well as the number of sampled alleles and allelic richness for forest (F) and viniculture (V) populations. Physically unlinked loci are marked with an asterisk (see Cano et al., 2011).

Locus	BFG130*	BFG092*	BFG066	BFG151	BFG090*	BFG082	BFG099*	BFG160*	BFG145*	BFG129
Motif	TCTT	TATC	AAG	GAAA	CTAT	TATC	ACTC	TCTA	TCTA	CTAT
AS [%]	100	84	87	93	78	96	99	100	96	96
**Number of alleles sampled**										
F	7	22	17	20	16	21	5	23	16	25
V	7	19	13	23	13	22	4	23	15	23
**Allelic richness**										
F	6.924	21.759	16.195	19.762	16.000	20.665	4.928	22.578	15.928	24.638
V	6.914	17.635	12.952	20.738	13.000	19.900	4.000	19.992	14.115	21.513

The main concern of larvae sampling is a potential bias of the results due to siblings in the data set. Removing full-siblings most likely produces results that are closer to those calculated from adult individuals and therefore improves the inference of population genetic studies based on larval samples (Goldberg & Waits, 2010). We removed full-siblings from the data by randomly selecting one tadpole per clutch, resulting in seven to ten individuals per population. We calculated Hardy-Weinberg-Equilibrium over all populations using GenePop 4.2 (Raymond & Rousset, 2004). We grouped individuals from populations P1–P6 into a viniculture population (V) and individuals from P7 to P10 into a forest population (F) and calculated the number of sampled alleles (N_A_) and allelic richness (N_AR_) using FSTAT 2.9.3.2 (Goudet, 2002).

The removal of full-siblings from data may improve the quality of the results, but causes a low number of individuals per site, especially in small populations. This might introduce a bias due to picking one individual over another. To compensate for this potential bias, we applied the repeated randomized selection of genotypes (RRSG) approach (Lenhardt & Theissinger, 2017). This approach for removing full-siblings from an offspring data set produces population estimates which are closer to estimates calculated for the parental data set, compared to estimates based on data containing siblings. Any potential bias due to selection of one sibling over another is compensated by performing multiple estimates of the genetic parameters. This RRSG approach was thus applied in all subsequent population genetic analyses.

To examine the genetic structure of the sampled populations, the Bayesian clustering software STRUCTURE 2.3.4 (Pritchard, Stephens & Donnelly, 2000) was used. Since the presence of siblings can also bias the detection of genetic clusters (Anderson & Dunham, 2008; Rodriguez-Ramilo & Wang, 2012), we again applied a RRSG approach creating 500 subsets of genotypes without siblings, resulting in 71 individuals from the populations P1–P9 per subset. Population P10 was excluded due to a possible isolation by distance effect (see results; Pritchard, Wen & Falush, 2010).

As we expected some genetic exchange between populations, but an overall weak population structuring, we chose the admixture model with imposed sampling locations (LOCPRIOR). The model was calculated with an initial burn-in of 100, 000 and a Markov Chain Monte Carlo (MCMC) of 500, 000 repeats for each subset and each predefined cluster number K between 1 and 9. To determine the most likely number of clusters K, the program STRUCTURE HARVESTER (Earl & VonHoldt, 2012) was used. Results were combined with the LargeKGreedy algorithm with 10, 000 random input orders in CLUMPP (Jakobsson & Rosenberg, 2007) and visualised with DISTRUCT (Rosenberg, 2004).

For linkage disequilibrium over all populations, population pairwise *F*_ST_ and *R*_ST_ as well as for observed (*H*_*O*_) and expected (*H*_*E*_) heterozygosity calculations we applied the RRSG approach with 100, 000 calculations using GenePop. Only one individual genotype per clutch was automatically selected in each calculation, thus producing results for linkage disequilibrium, *F*_ST_, *R*_ST_, *H*_*O*_ and *H*_*E*_ values based on data without full-siblings. For interpretation, we used median pairwise *F*_ST_ (MPF) and median pairwise *R*_ST_ (MPR) values as well as median *H*_*O*_ and *H*_*E*_ values over all RRSG calculations. For the interpretation of the linkage disequilibrium we, calculated a possibility of linkage for each loci pair by forming a quotient of number of calculations where linkage was detected (*p*-value ≤0.05) divided by total number of calculations of the RRSG approach. We considered a loci pair linked when 5% or more of the 100, 000 calculations detected a statically significant linkage disequilibrium for the respective loci pair.

We calculated a distance matrix for the breeding ponds and analyzed isolation by distance for MPFs and MPRs over all breeding pond pairs using Genepop’s subprogram ISOLDE (Rousset, 2008). We used MPF/(1-MPF) and MPR/(1-MPR) as the dependent variable and the corresponding linear geographic distance, number of roads as well as the cumulated traffic intensity of all roads (vehicles per 24 h; received from the Ministry of the Interior, Sports and Infrastructure Rhineland-Palatinate in 2015; Tables S1 and S2) between breeding ponds as the independent variable in a Mantel’s test with Spearman rank correlation for matrix correlation with 10, 000 permutations (Rousset, 1997).

To address the spatial configuration of habitat types between breeding ponds, we adjusted the linear geographic distance with respect to present habitat types. Therefore, we obtained land cover data (ATKIS) of the study area from the State Office for Surveying and Geobasisinformation Rhineland-Palatinate (2015). We calculated the area of habitat types (settlements, viniculture, grassland, meadows, copse, forest and waterbodies) and length of roads in a 200 m wide strip between breeding ponds. Since the vinicultural study area has, apart from of the ponds and their surrounding areas, no mentionable hideout and hibernation options for amphibians, we limited our analysis of the spatial configuration to the most direct migration routes for amphibians between ponds. Assuming an average daily migration distance of 100 m (Berger, Pfeffer & Kalettka, 2011), 200 m wide strips take possible deviations from this average daily migration distance, resulting for example from foraging, into account (see also Vos et al., 2001; Arens et al., 2007).

Positive habitat types like grassland, meadows, copse, forest and waterbodies may increase the daily migration distance of amphibians due to favorable migration conditions (such as food availability, humidity and protection against predators). On the other hand, negative habitat types like settlements and viniculture may decrease the daily migration distance due to unfavorable migration conditions. In a weighted distance model, such positive and negative effects of habitat types on the migration of amphibians between breeding ponds can be addressed. We adapted a weighted distance model (Vos et al., 2001; Arens et al., 2007), which corrects the linear geographic distance based on the negative and positive habitat types between breeding ponds. We introduced a habitat correction factor into the model (Table 2), since each habitat type may impact the genetic differentiation with a different magnitude. For each habitat type, we calculated the corrected linear geographic distance using the weighted distance model with a habitat correction factor from 1 to 100 in steps of 0.1. We selected the relevant habitat correction factor based on the highest *R*^2^ of MPF as well as MPR and the corrected linear geographic distance. Afterwards, we used ISOLDE to analyze isolation by distance for MPFs as well as MPRs and the corrected linear geographic distance with the relevant habitat correction factor provided by the weighted distance model, for each habitat type separately. Finally, we combined all habitats (see Tables 2 and 3) that showed statistically significant isolation by distance in the individual weighted distance models into one weighted distance model and analyzed isolation by distance for MPFs as well as MPRs using ISOLDE.

**Table 2:** Overview of all weighted distance models.

Weighted distance models	Description
LGD*R_NA_	Linear geographic distance (LGD) weighted for the fraction of negative area (NA). R_NA_ being the negative area relative to the total area (TA) in a strip of 200 m wide between two ponds. Adjusted with the habitat correction factor (HCF)
	R_NA_ = (NA∗HCF + TA)∕TA
LGD*R_PA_	Linear geographic distance (LGD) weighted for the fraction of positive area (PA). R_PA_ being the positive area relative to the total area (TA) in a strip of 200 m wide between two ponds. Adjusted with the habitat correction factor (HCF)
	R_PA_ = TA∕(PA∗HCF + TA)
LGD*R_NA_*R_PA_	Combined weighted distance for positive and negative area.

**Table 3:** Results of isolation by distance for median pairwise *F*_ST_ (MPF) as well as median pairwise *R*_ST_ (MPR) and the linear geographic distance (LGD) corrected by the weighted distance models with habitat correction factor (HCF).

	MPFs			MPRs		
Weighted distance model	HCF	*p*-value	*R*^2^	HCF	*p*-value	*R*^2^
LGD*R_NA_ viniculture	10.8	<0.001	0.327	7.3	0.008	0.159
LGD*R_NA_ settlements	88.5	0.125	0.107	1.0	0.153	0.040
LGD*R_PA_ forest	8.8	0.005	0.303	4.0	0.016	0.079
LGD*R_PA_ grassland	16.2	0.365	0.043	38.5	0.239	0.069
LGD*R_PA_ meadows	11.6	0.165	0.302	10.3	0.092	0.140
LGD*R_PA_ copse	1.0	0.288	0.031	1.0	0.143	0.040
LGD*R_PA_ waterbodies	97.0	0.316	0.038	1.0	0.137	0.041

## Results

We detected deviation from Hardy-Weinberg-Equilibrium due to heterozygote deficits on two loci (BFG082 and BFG129) over all populations. Forest populations showed higher values for number of sampled alleles and allelic richness in comparison to population viniculture (Table 1). Over all populations, we detected linkage disequilibrium for 27 out of 45 loci pairs (see Table S3). The highest percentage of linkage disequilibrium was detected for the locus pair BFG66 & BFG90 (95%). Also, we detected linkage disequilibrium for loci pairs that are physically unlinked (i.e., located on different chromosomes, Cano et al., 2011), for example BFG90 & BFG145 (86%), BFG90 & BFG160 (78%) and BFG92 & BFG145 (68%).

**Figure 2:**
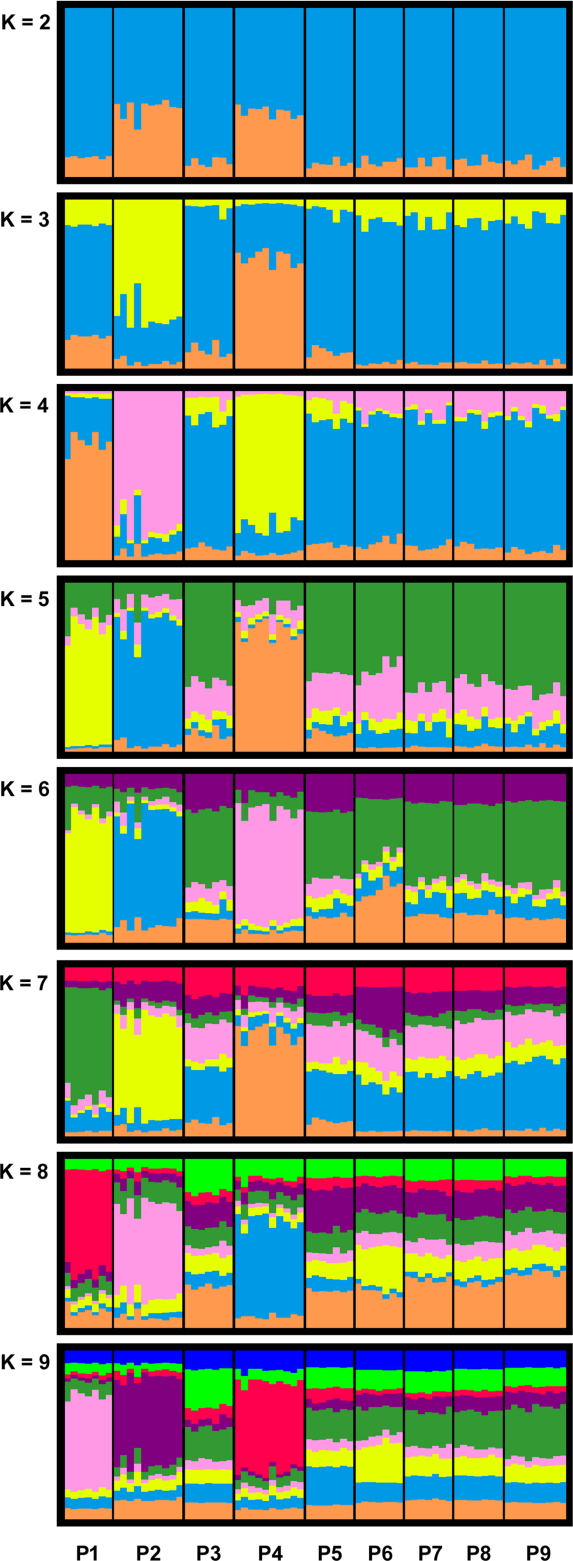
Bar plots of combined STRUCTURE analysis for clusters *K* = 2 to *K* = 9 of the investigated *R. temporaria* breeding pond populations in the study area. STRUCTURE HARVESTER identified *K* = 4 as the most meaningful number of clusters. Each vertical bar represents one individual, and the color composition visualizes the probability to belong to one of the K clusters defined by STRUCTURE. P10 was excluded from the analysis due to the different life stage of the samples.

STRUCTURE HARVESTER identified *K* = 4 as the most meaningful number of clusters in our data set (see Fig. S5 and Table 4). For *K* = 4, we detected for the breeding pond populations P1, P2 and P4 separate clusters, whereas the remaining populations formed a joined cluster. With an increased *K* (*K* = 5 to *K* = 9), P1, P2 and P4 still formed individual clusters, while the rest of the populations where assigned to the same cluster up to *K* = 7 (Fig. 2).

**Table 4:** Expected and observed heterozygosity calculated with the repeated randomized selection of genotypes (RRSG) approach over all loci for breeding pond populations P1–P10.

	**P1**	**P2**	**P3**	**P4**	**P5**	**P6**	**P7**	**P8**	**P9**	**P10**
*H*_*E*_	0.852	0.685	0.776	0.722	0.703	0.664	0.788	0.831	0.840	0.824
*H*_*O*_	0.757	0.600	0.643	0.560	0.629	0.514	0.657	0.771	0.778	0.738

**Table 5:** Results of the repeated randomized selection of genotypes (RRSG) approach for the median pairwise *F*_ST_ (MPF) and median pairwise *R*_ST_ (MPR). Populations 1–6 were located within vineyards, populations 7–10 in the Palatinate Forest. Population 10 was about 40 km away from the core study area.

	MPR									
	Pop.	1	2	3	4	5	6	7	8	9	10
MPF	1	–	0.1137	0.0104	0.0471	0.0022	0.0333	0.0518	0.0449	0.0826	0.1403
	2	0.0987	–	0.0851	0.0854	0.0577	0.0866	0.0277	0.0221	0.0000	0.0607
	3	0.0559	0.0523	–	0.0006	0.0000	0.0016	0.0005	0.0405	0.0333	0.0176
	4	0.0802	0.0781	0.0372	–	0.0000	0.1027	0.0975	0.0471	0.0355	0.0872
	5	0.0532	0.0519	0.0215	0.0457	–	0.0108	0.0536	0.0018	0.0260	0.0640
	6	0.0672	0.0383	0.0224	0.0540	0.0268	–	0.0093	0.0537	0.0572	0.0607
	7	0.0575	0.0387	0.0223	0.0479	0.0266	0.0012	–	0.0000	0.0000	0.0000
	8	0.0574	0.0418	0.0179	0.0459	0.0191	0.0123	0.0064	–	0.0000	0.0451
	9	0.0441	0.0339	0.0135	0.0410	0.0084	0.0075	0.0159	0.0041	–	0.0103
	10	0.0687	0.0708	0.0328	0.0751	0.0434	0.0374	0.0409	0.0212	0.0265	–

With exception of P1 (*H*_*E*_ = 0.852), all pond populations located in viniculture showed lower levels of heterozygosity over all loci (*H*_*E*_ = 0.663–0.776) than populations located in the Palatinate Forest (P7–P10; *H*_*E*_ = 0.788–0.840; Table 4). MPFs ranged from 0.0012 to 0.0987 and MPRs from 0.0000 to 0.1403 (Table 5). The highest MPF and MPR were estimated between P1 and P2 at a linear geographic distance of 2.4 km (see Table S5 for a matrix of all linear geographic distances). The lowest MPF was found between P6 and P7 at a linear geographic distance of 7.9 km. On average, genetic differentiation between population pairs in viniculture (average MPF = 0.0523, average MPR = 0.0425) was higher than between population pairs in forest or forest and viniculture, whereas population pairs in the Palatinate Forest showed the lowest MPFs and MPRs (average MPF = 0.0192, average MPR = 0.0092). In general, genetic differentiation among breeding pond populations in viniculture was comparatively high, despite close proximity of the breeding ponds (e.g., linear geographic distance <1 km: MPF = 0.0467; linear geographic distance <2.5 km: MPF = 0.0987 and MPR = 0.1027), as opposed to breeding pond populations in the forest (linear geographic distances = 4.5–9.5 km; MPFs = 0.0064–0.0409 and MPRs = 0.0041–0.0648). Yet populations at breeding ponds with a direct connection to the Palatinate Forest by permanent or seasonal streams exhibited lower MPFs to forest pond populations (P3, P5 and P6) compared with agricultural pond populations not connected to the forest (P1, P2 and P4; see Table 5).

Over all breeding pond populations, ISOLDE detected no statistically significant relation between MPFs or MPRs and linear geographic distance, number of roads or accumulated traffic intensity between population pairs (*p* > 0.050). Isolation by distance was statistically significant for MPFs of the four forest populations (*p* = 0.0320). However, when excluding the most distant population P10, isolation by distance was no longer statistically significant.

When analyzing the linear geographic distances corrected by the weighted distance model, isolation by distance was statistically significant (*p*-values < 0.050) for viniculture and forest (MPF and MPR; Table 3). Corrected linear geographic distances of viniculture, forest and meadows showed an explained variance of more than 0.300 when correlated with MPF, whereas explained variance was significantly lower when correlated with MPR (0.079–0.159). Combining all distance corrections (R_NA_ viniculture and R_PA_ forest, see Tables 2 and 3) that showed statistically significant isolation by distance into one weighted distance model resulted in statistically significant isolation by distance for MPF as well as MPR (*p*-values < 0.005).

## Discussion

We analyzed the genetic differentiation of *R. temporaria* of breeding pond populations within viniculture and the Palatinate Forest to investigate potential genetic population differentiation due to agricultural land use. Our microsatellite data exhibited linkage disequilibrium for 27 of the 45 loci pairs. However, high percentages (up to 95%) of linkage disequilibrium were also detected for multiple loci pairs located on different chromosomes and for which linkage is thus unlikely. Moreover, the linkage calculations were performed over the whole dataset as one metapopulation. This could have additionally affected the linkage analyses due to the underlying population structuring, since specific allele combinations might only occur in some fragmented populations, thus inferring linked inheritage of respective loci. The vice versa assumption, that genetically linked loci might have inferred the detected population fragmentation by structure as unreal signal in our data, can be rejected, since our analyses for gene flow among all populations (MPFs and MPRs, Table 5) also suggested that the fragmented populations P1, P2 and P4 were more isolated compared to the other populations. Thus, we evaluated the detected linkage disequilibrium as statistical artefact and decided to use all ten loci for subsequent analyses.

Our analysis showed structuring within the investigated breeding pond populations and highlighted breeding pond populations P1, P2 and P4 (all located in viniculture) as isolated from the meta-population (Fig. 2). Moreover, our data exhibited higher genetic differentiation among breeding pond populations in the agricultural landscape compared with breeding pond populations in the Palatinate Forest (Table 5). We observed the highest genetic differentiation between breeding pond populations in viniculture, which were only a few kilometers apart (e.g., P1 and P2 with a linear geographic distance of less than 2.5 km: MPF = 0.0987 and MPR = 0.1137). The most distant forest population P10 was responsible for a significant isolation by distance among the forest populations. However the results for P10 have to be treated with caution, since we mixed different life stages and generations, which may introduce some bias (Peterman et al., 2016). Still, even when we exclude P10 from the data set, the genetic differentiation within the remaining forest populations was lower compared with viniculture populations.

Breeding pond populations in the agricultural landscape with a direct connection to the Palatinate Forest by permanent or seasonal streams exhibited lower MPFs to forest pond populations compared with agricultural pond populations not connected to the forest (Table 5), indicating the importance of waterbodies including the adjacent riparian vegetation for the genetic connectivity in amphibian breeding pond populations. In 2012, we observed the translocation of *Rana temporaria* clutches at P8, which were intentionally moved into the nearby stream due to drought by staff of the “Modenbacher Hof”, a close-by horse ranch. This stream is connected directly to P3. During major rain events, some of the clutches could have been flushed into the pond at P3. Surviving amphibians could then have contributed to the following reproduction phases, resulting in a one directional genetic exchange and explaining the rather low MPF value of 0.0179 between P3 and P8.

Our population genetic results were similar to the differentiation of *Rana arvalis* (*F*_ST_ = 0.06) in Noord-Brabant, Netherlands, where landscape permeability was low due to farming intensity and urbanization (Van der Sluis & Vos, 1997; Arens et al., 2007). Additionally, breeding sites in Noord-Brabant became polluted with agrochemicals (pesticides and fertilizers) as a result of intensive agriculture (Hoogerwerf & Crombaghs, 1993). For *R. temporaria*, Safner et al. (2011) found *F*_ST_ values between 0.024 and 0.193 in a human dominated landscape near Chambery, France, on a fine spatial scale (<20 km). Negative effects of high agricultural intensity on the occurrence, abundance and genetic diversity of amphibians on a regional and national scale were also found in several other studies (Johansson et al., 2005; Trochet et al., 2016; Youngquist et al., 2016).

Our analyses in ISOLDE rejected isolation by distance based on roads and associated traffic intensity as the sole explanation of the genetic differentiation of *R. temporaria*; although, an effect of roads on amphibian population connectivity has been shown in other studies (Buskirk, 2012; Beebee, 2013; Krug & Pröhl, 2013). However, the weighted distance model showed significant isolation by distance for viniculture and forest, indicating that these two habitat types are the most relevant parameters to explain the structuring of breeding pond populations in the study area. Also, the introduction of the habitat correction factor to the weighted distance model showed that applying habitat specific permeability can improve the detection of isolation by distance remarkably. However, the habitat correction factor has to be interpreted in context with the explained variance in the isolation by distance analyses, since a high habitat correction factor not necessarily translates into a high impact on population differentiation when explained variance is low (<0.1). With exception of habitat type copse, introducing the habitat correction factor to the weighted distance model did improve the explained variance of the corrected linear geographic distance, when correlated with MPF. For MPR, settlements, copse and waterbodies did not benefit from the introduction of the habitat correction factor.

Lenhardt et al. (2013) assessed the potential fragmentation of breeding sites in the same study area with a simplified expert based landscape permeability model. They predicted fragmentation, and therefore a potential genetic differentiation, of agricultural breeding ponds in close proximity, when pesticide applications were considered as a migration limiting model factor. Our genetic data presented here confirmed the predicted fragmentation of P1 from the other breeding pond populations (MPFs from 0.0553 to 0.0987; Table 5). However, the model in Lenhardt et al. (2013) overestimated the potential fragmentation of breeding sites in a number of cases, especially when the breeding ponds in viniculture (e.g., P3 and P6, Fig. 1) were directly connected to the Palatinate Forest via permanent streams. Thus, permanent streams and their riparian vegetation may serve as suitable migration or dispersal corridors within the agricultural landscape.

In our study area, the intensification of viniculture started in the early 20th century. Particularly in the last 50 to 80 years, the development of mechanical equipment and the broad availability of pesticides have led to a further intensification and expansion of viniculture, leaving amphibian species like *Rana temporaria* with small fragmented breeding habitats within the agricultural landscape. Nowadays, typical application scenarios in vineyards of Southern Palatinate consist of up to 12 (on average 8) fungicide applications per year, within intervals of about 10–14 days between early May and mid-August (Roßberg, 2009; Lenhardt et al., 2013). During this period, amphibians are in their terrestrial life stage and juvenile individuals migrate away from the spawning waters. Furthermore, fungicide applications are often applied before or after rain events of more than 3 mm precipitation (Lenhardt et al., 2013). Such rain events may trigger amphibian migration and general amphibian activity (Rothermel, 2004; Baldwin, Calhoun & DeMaynadier, 2006). Therefore, the spatial and temporal overlap of amphibians and applied fungicides is very likely.

Since *R. temporaria* becomes sexually mature in the third (rarely second or first) year of life (WestheideRieger, 2015), about 25–40 overlapping generations have passed since the intensification of viniculture started. Due to the few passed generations, overall population differentiation is still moderate (*F*_ST_ between 0.05 –0.15; Hartl & Clark, 2007; Wright, 1978) but may increase due to time-delay in genetic differentiation (Bossart & Pashley Prowell, 1998). Also, *F*_ST_ might already underestimate the current genetic differentiation when polymorphic loci are used in highly structured populations, since *F*_ST_ can’t distinguish between mutation and dispersal (Balloux & Lugon-Moulin, 2002). The genetic differentiations identified by MPF values were supported by the estimated MPR values (Table 5), which underlines a separation of breeding pond populations in the study area.

Due to the temporal coincidence of amphibian activity and pesticide applications, negative effects on meta-population dynamics could be expected in a viniculture landscape, if fungicides are generally of high toxicity and exposure of amphibians is high. Also, pesticide applications were the most frequent management measures in viniculture (up to 12 applications) and can affect amphibians not only on the application day, like tillage operations, but up to several days after application, depending on the chemical decomposition of pesticides. Recent studies and surveys confirmed the presence of pesticides in amphibian habitats and waterbodies in general (Smalling et al., 2012; Ulrich et al., 2015), as well as in amphibian tissues (Smalling et al., 2013; Smalling et al., 2015; Battaglin et al., 2016; Cusaac et al., 2016). Furthermore, pesticide concentrations in amphibian tissues were positively correlated with agricultural and urban land around breeding sites (Battaglin et al., 2016). Therefore, pesticides may be a major factor for the detected genetic differentiation within the investigated *R. temporaria* breeding pond populations. Yet we can only assume this impact and want to highlight the need of more detailed studies on the effects of pesticides on natural amphibian populations, taking different life stages as well as different species into account.

We were not able to address differences between organic and conventional viniculture, since reference breeding sites with noteworthy portions of organic viniculture were not available in or nearby the study area. Also, it is currently unclear if the use of copper and sulfur within organic viniculture would actually improve the overall situation for amphibians (Mackie et al., 2013; Milanovi, Comitini & Ciani, 2013).

In contrast to our and others findings, some studies observed no impact of agricultural land use on the genetic differentiation of amphibians, although the investigated amphibian species were known to forage in intensively managed agricultural areas (Le Lay et al., 2015; Frei et al., 2016). Also, some level of pesticide tolerance for amphibians from agricultural breeding pond populations was detected (Hua, Morehouse & Relyea, 2013; Hua et al., 2015). Yet such findings should not be generalized, since tested taxa and pesticides were limited, and pesticides still may cause lethal or sublethal effects on amphibians, depending on the path of exposure, exposure level and amphibian life stage.

Although *R. temporaria* is considered ‘not endangered’ in Germany (Kühnel et al., 2009) and ‘least concerned’ in Europe (Temple & Cox, 2009), amphibian census indicated that many breeding pond populations, especially in agricultural land, were rather small (one to ten clutches) and populations with more than 150 clutches were generally rare (Schlüpmann, Schulze & Meyer, 2004; Schlüpmann et al., 1996; Wolfbeck, Laufer & Genthner, 2007). Consistent with these observations, amphibian surveys in the study area counted between 1 and 60 clutches per breeding site during 2007–2010 (S Bischoff, pers. comm., 2011; Table S6). We repeatedly counted ten or less clutches for all breeding pond populations within viniculture (P1–P6) during our samplings from 2012 to 2014. Considering the small size of breeding pond populations in viniculture, local extinction may occur when breeding sites have a loose connectivity to surrounding terrestrial habitats (Safner et al., 2011).

Based on our results, we are concerned about the persistence of amphibians in agricultural areas, since we can recognize negative trends on the genetic diversity and differentiation of breeding pond populations. Typical visible barriers like roads with associated amphibian road mortality could not explain the genetic structuring of the breeding sites. Yet we could identify viniculture as a barrier for genetic exchange. Since pesticide applications are the most frequent management measure in viniculture and pesticides can cause high mortalities in amphibians, pesticides may have a major impact on amphibian dispersal and therefore on genetic exchange between breeding sites. Following the precautionary principle it may be advisable to reduce or avoid pesticide applications during amphibian migration phases and to mitigate pesticide contamination of amphibian breeding ponds. We recommend further research on the impact of pesticides on amphibian individuals and populations in agricultural landscapes.

##  Supplemental Information

10.7717/peerj.3520/supp-1 Supplemental Information 1 Appendix A

## Additional Information and Declarations

### Field Study Permissions

The following information was supplied relating to field study approvals (i.e., approving body and any reference numbers): Field experiments were approved by the ”Struktur- und Genehmigungsdirektion Süd Referat 42- Obere Naturschutzbehörde” (Approval Number: 42/553-254).

### Data Availability

The following information was supplied regarding data availability: The raw data has been supplied as Supplemental Information 1.
