# Supplementary material for: Amphibian population genetics in agricultural landscapes: does viniculture drive the population structuring of the European common frog (Rana temporaria)?
Source: PeerJ. 2017 Jul 11;5:e3520. doi: 10.7717/peerj.3520 (PMC5508807; doi:10.7717/peerj.3520)
Supplement: Supplemental Information 1 [file peerj-05-3520-s001.pdf]

Appendix

Amphibian population genetics in agricultural landscapes: Does viniculture drive the population structuring of the European common frog (*Rana temporaria*)?

Patrick P. Lenhardt, Carsten A. Brühl, Christop Leeb, Kathrin Theissinger

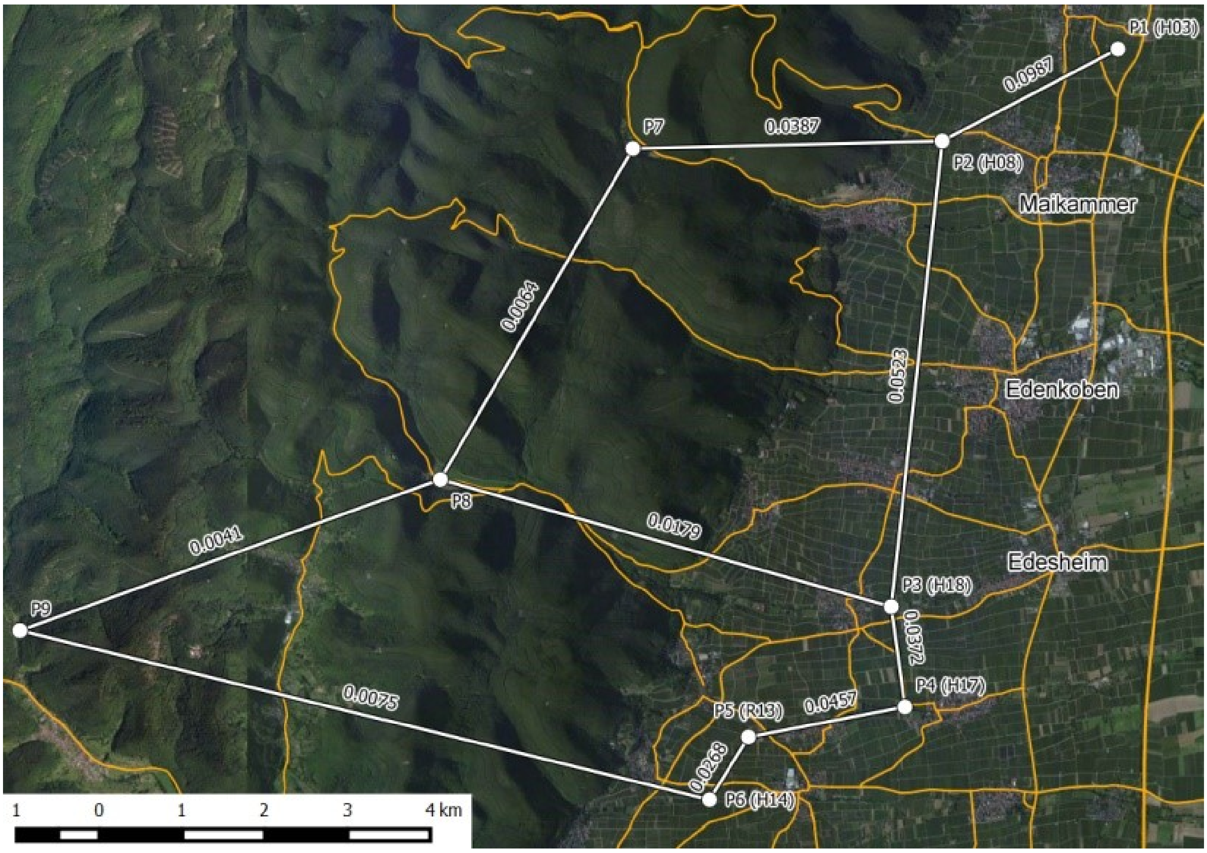

Appendix Figure 1. Aerial photo of the core study area between "Neustadt (an der Weinstraße)" (north of P1) and "Landau (in der Pfalz)" (south of P6) with selected median pairwise F_{ST} values. Relevant traffic infrastructure is highlighted in orange (aerial photo from Bing Maps, http://www.bing.com/mapspreview).

Appendix

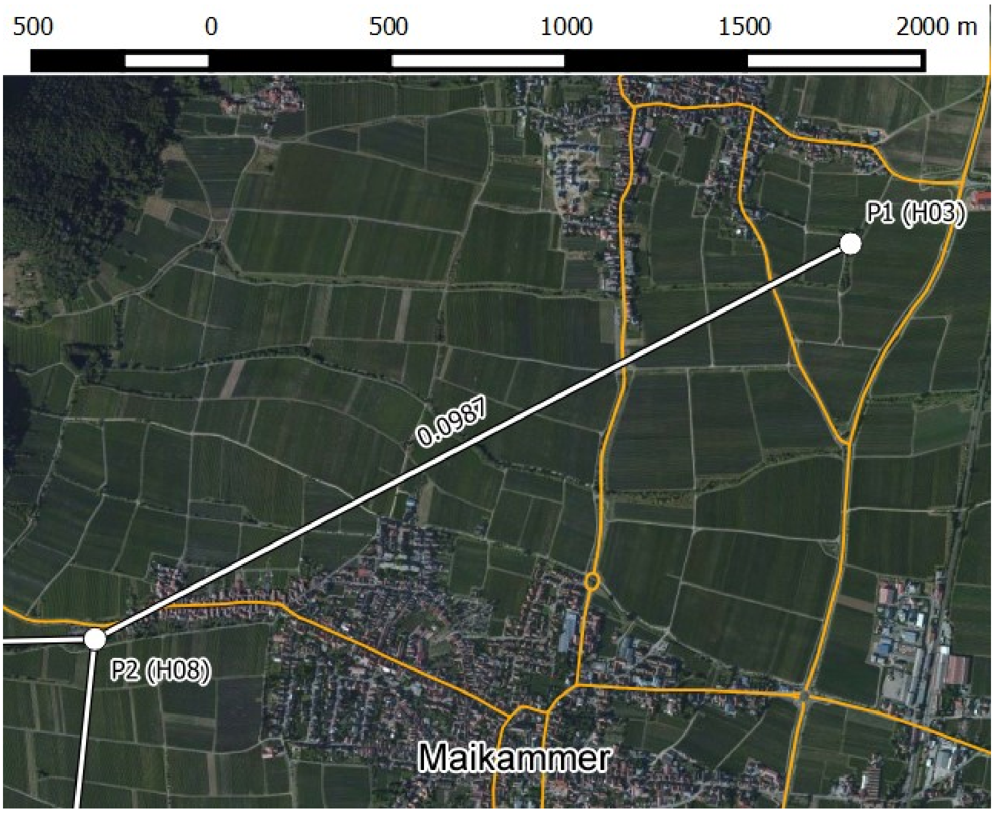

Appendix Figure 2. Close up aerial photo of landscape between breeding ponds P1 and P2 (2 394 m apart) in the north of the core study area with median pairwise F_{ST} value. Relevant traffic infrastructure is highlighted in orange (aerial photo from Bing Maps, http://www.bing.com/mapspreview).

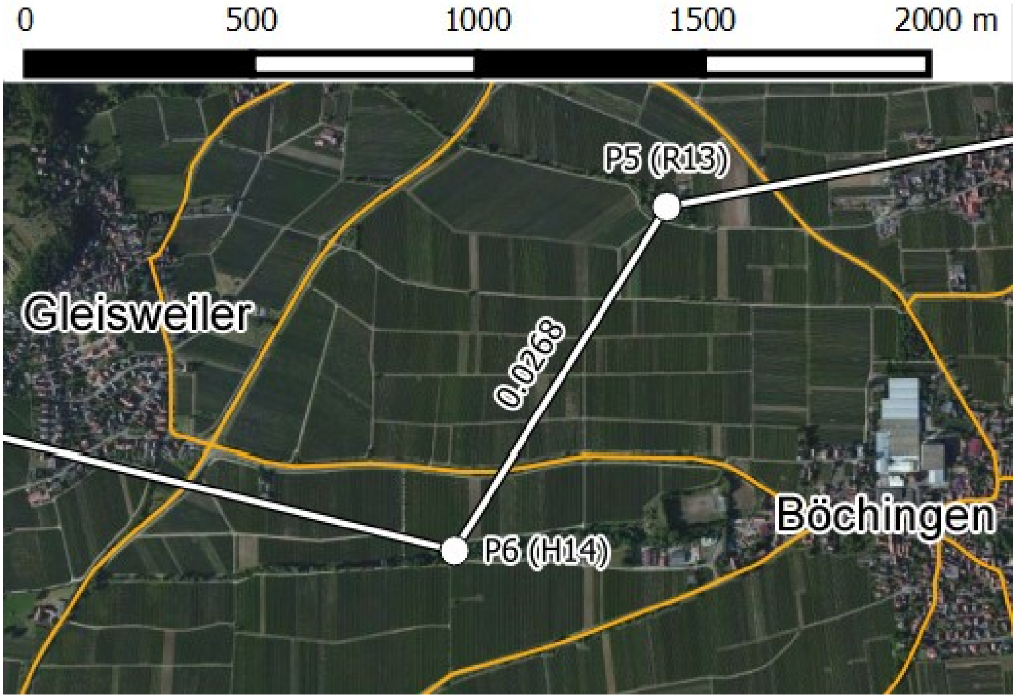

Appendix Figure 3. Close up aerial photo of landscape between breeding ponds P5 and P6 (890 m apart) with median pairwise F_{ST} value. Relevant traffic infrastructure is highlighted in orange (aerial photo from Bing Maps, http://www.bing.com/mapspreview).

Appendix

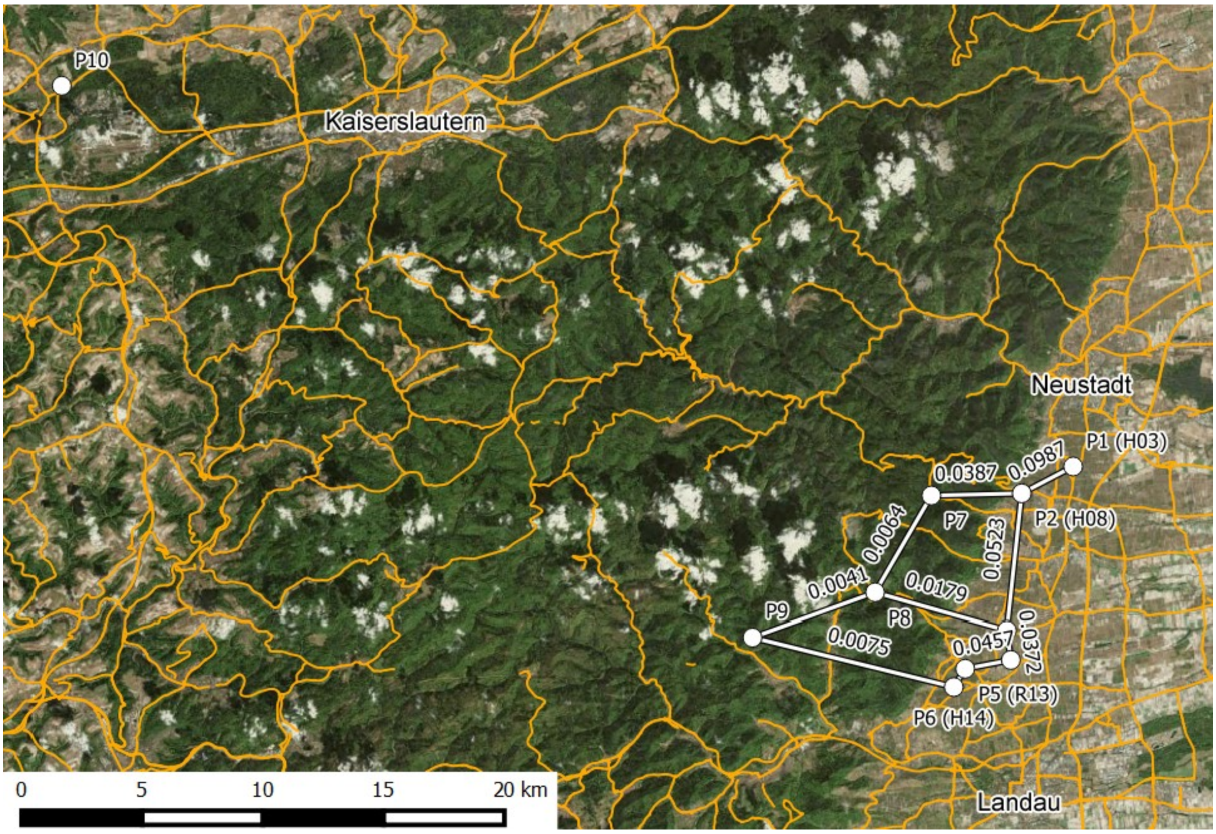

Appendix Figure 4. Aerial photo of the core study area between between "Neustadt (an der Weinstraße)" and "Landau (in der Pfalz)" with selected median pairwise F_{ST} values and P10 near Kaiserslautern. Relevant traffic infrastructure is highlighted in orange (aerial photo from Bing Maps, http://www.bing.com/mapspreview).

Appendix

Appendix Table 1. Overview of all relevant roads in the study area. Traffic intensity (vehicles per 24 hours) were obtained from the Ministry of the Inner, Sports and Infrastructure in Rhineland-Palatinate (marked with an asterisk; Iris Honrath, personal communication) or estimated based on traffic intensity of nearby roads and geographical location.

Road	Type	Traffic (in 24h)	
A 6	motorway	62674	*
B 270	primary	14280	*
L 395	secondary	6000	
L 502	secondary	6000	
L 503	secondary	6259	*
L 369	secondary	6000	
L 356	secondary	6000	
K 32	tertiary	3148	*
K 5	secondary	3000	
K 53	tertiary	3000	
K 50	tertiary	3000	
B 48	primary	2934	*
L 506	secondary	2836	*
L 514	secondary	2632	*
L 499	secondary	2549	*
L 512	secondary	2500	
L 500	secondary	2000	
L 519	secondary	1955	*
L 513	secondary	1553	*
K 49	tertiary	1500	
K 55	tertiary	1500	

Road	Type	Traffic (in 24h)	
K 15	secondary	1500	
K 4	tertiary	1500	
L 505	secondary	1500	
L 507	secondary	1456	*
L 504	secondary	1217	*
K 57	tertiary	1067	*
K 40	secondary	1000	
K 38	tertiary	1000	
K 17	tertiary	1000	
K 18	tertiary	1000	
K 19	tertiary	1000	
K 51	unclassified	1000	
K 30	unclassified	931	
K 31	secondary	931	
K 6	tertiary	931	*
K 58	tertiary	729	*
K 59	tertiary	729	
L 515	secondary	645	*
K 78	unclassified	586	
K 56	tertiary	586	*

Appendix

Appendix Table 2. Number of roads between population pairs and their accumulated traffic intensity (vehicles in 24 hours on all roads between the pairs).

		P1	P2	P3	P4	P5	P6	P7	P8	P9
Number of roads	P2	3								
	P3	7	6							
	P4	10	7	2						
	P5	9	8	4	2					
	P6	10	9	5	3	1				
	P7	4	2	3	5	5	7			
	P8	6	3	4	7	5	5	1		
	P9	6	4	3	4	3	4	1	2	
	P10	14	15	15	18	15	15	15	12	12
accumulated traffic intensity	P2	4645								
	P3	14192	12978							
	P4	17165	14089	2042						
	P5	16715	13730	5609	3567					
	P6	18268	15283	7162	5120	1553				
	P7	7277	3277	6267	8309	7019	10189			
	P8	9139	4208	6996	9767	8245	8731	931		
	P9	11044	7044	6065	7521	6247	7800	931	3767	
	P10	122690	123467	119481	124252	115788	116341	121691	109886	109955

Appendix

Appendix Table 3 Linkage of loci pairs with RRSG approach. Percentage values indicate the relative number of runs out of 100 000 calculations where linkage was detected (p-value less or equal to 0.05).

Loci pair	Linkage detected out of 100 000 calculations [%]	Loci pair	Linkage detected out of 100 000 calculations [%]
BFG130 & BFG092	0,04	BFG130 & BFG066	0,00
BFG090 & BFG129	0,72	BFG130 & BFG099	0,00
BFG090 & BFG145	0,87	BFG130 & BFG151	0,00
BFG090 & BFG082	0,34	BFG130 & BFG160	0,00
BFG090 & BFG099	0,00	BFG066 & BFG090	0,96
BFG090 & BFG160	0,08	BFG066 & BFG129	0,64
BFG145 & BFG129	0,78	BFG066 & BFG145	0,18
BFG082 & BFG129	0,40	BFG066 & BFG082	0,00
BFG082 & BFG145	0,65	BFG066 & BFG099	0,02
BFG082 & BFG099	0,00	BFG066 & BFG151	0,00
BFG082 & BFG160	0,01	BFG066 & BFG160	0,04
BFG092 & BFG090	0,74	BFG099 & BFG129	0,05
BFG092 & BFG129	0,70	BFG099 & BFG145	0,04
BFG092 & BFG145	0,69	BFG099 & BFG160	0,00
BFG092 & BFG082	0,42	BFG151 & BFG090	0,59
BFG092 & BFG066	0,24	BFG151 & BFG129	0,57
BFG092 & BFG099	0,00	BFG151 & BFG145	0,81
BFG092 & BFG151	0,74	BFG151 & BFG082	0,18
BFG092 & BFG160	0,13	BFG151 & BFG099	0,22
BFG130 & BFG090	0,00	BFG151 & BFG160	0,12
BFG130 & BFG129	0,13	BFG160 & BFG129	0,59
BFG130 & BFG145	0,19	BFG160 & BFG145	0,30
BFG130 & BFG082	0,02		

Appendix

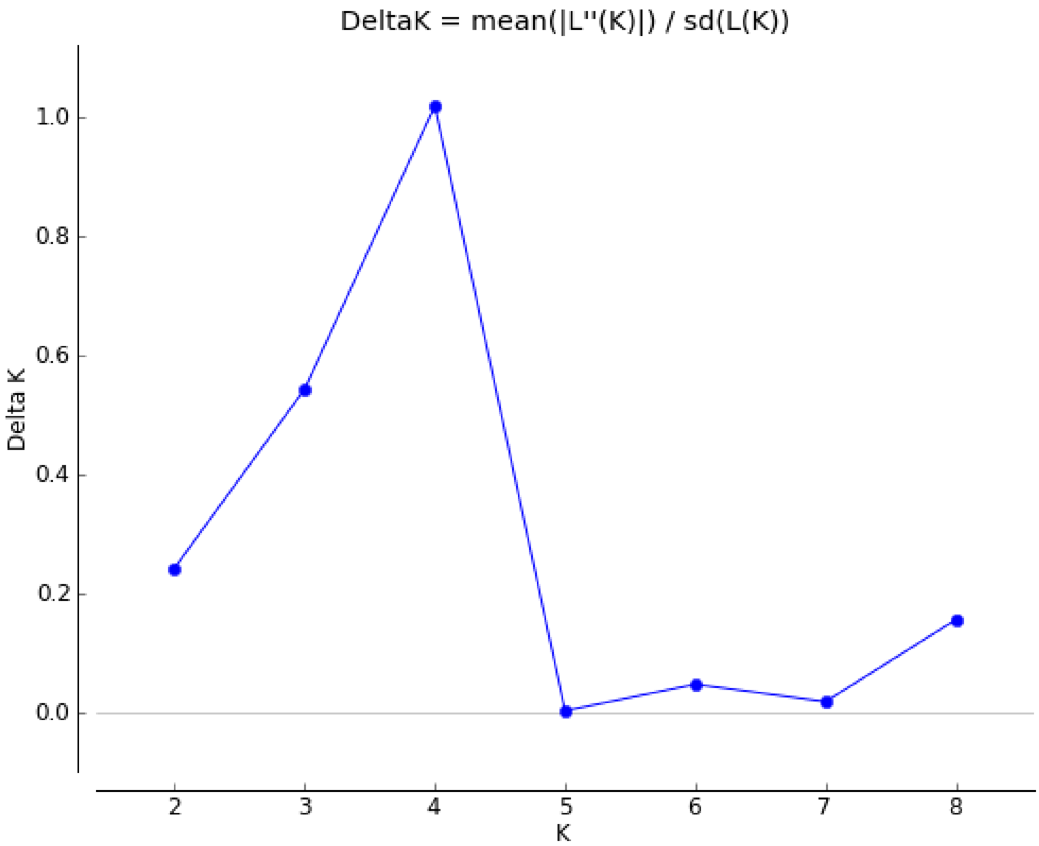

Appendix Figure 5. Plot of delta K values from the Structure analyses, obtained through STRUCTURE HARVESTER.

Appendix Table 4 Evanno table output from the Structure analyses, obtained through STRUCTURE HARVESTER.

K	Reps	Mean LnP(K)	Stdev LnP(K)	Ln'(K)	|Ln''(K)|	Delta K
1	500	-3327.7386	43.1840	NA	NA	NA
2	500	-3291.5598	49.5511	36.178800	12.029200	0.242764
3	500	-3267.4102	56.2733	24.149600	30.581800	0.543451
4	500	-3273.8424	86.4383	-6.432200	88.074400	1.018928
5	500	-3368.3490	139.3570	-94.506600	0.514200	0.003690
6	500	-3463.3698	172.8867	-95.020800	8.314400	0.048092
7	500	-3566.7050	205.5675	-103.335200	3.897000	0.018957
8	500	-3666.1432	309.9213	-99.438200	48.534200	0.156602
9	500	-3717.0472	337.7702	-50.904000	NA	NA

Appendix Table 5. Linear geographical distance (LGD) in km between all population pairs.

Appendix

LGD in km		P1	P2	P3	P4	P5	P6	P7	P8	P9
	P2	2.394								
	P3	7.254	5.644							
	P4	8.334	6.834	1.220						
	P5	9.415	7.553	2.335	1.926					
	P6	10.303	8.420	3.200	2.614	0.890				
	P7	5.979	3.737	6.342	7.489	7.227	7.901			
	P8	9.689	7.306	5.662	6.250	4.849	5.049	4.616		
	P9	14.992	12.598	10.526	10.728	8.890	8.573	9.403	5.387	
	P10	44.563	43.008	44.974	45.732	44.321	44.346	39.639	39.512	36.423

Appendix Table 6. Clutch counts at the monitored amphibian breeding sites with presence of Rana temporaria during breeding seasons 2011 to 2014 (na = not available).

Pop.	2011	2012	2013	2014
P1	8	7	7	0
P2	10	12	0	8
P3	9	11	8	0
P4	19	22	18	0
P5	7	8	0	0
P6	8	8	5	0
P7	26	32	27	24
P8	21	25	19	18
P9	> 100	> 100	> 100	> 100
P10	> 100	na	na	na
